# p38MAPK and Chemotherapy: We Always Need to Hear Both Sides of the Story

**DOI:** 10.3389/fcell.2016.00069

**Published:** 2016-06-30

**Authors:** Jesús García-Cano, Olga Roche, Francisco J. Cimas, Raquel Pascual-Serra, Marta Ortega-Muelas, Diego M. Fernández-Aroca, Ricardo Sánchez-Prieto

**Affiliations:** Unidad de Medicina Molecular, Laboratorio de Oncología, Centro Regional de Investigaciones Biomédicas, Unidad de Biomedicina UCLM-CSIC, Universidad de Castilla-La Mancha/PCTCLMAlbacete, Spain

**Keywords:** p38MAPK, cancer, chemotherapy, targeted therapy, resistance, sensitivity, p38MAPK inhibitors

## Abstract

The p38MAPK signaling pathway was initially described as a stress response mechanism. In fact, during previous decades, it was considered a pathway with little interest in oncology especially in comparison with other MAPKs such as ERK1/2, known to be target of oncogenes like Ras. However, its involvement in apoptotic cell death phenomena makes this signaling pathway more attractive for many cancer research laboratories. This apoptotic role allows to establish a link between p38MAPK and regular chemotherapeutic agents such as Cisplatin or base analogs (Cytarabine, Gemcitabine or 5-Fluorouracil) which are currently used in hospitals across the world. In fact, and more recently, p38MAPK has also been connected with targeted therapies like tyrosine kinase inhibitors (vg. Imatinib, Sorafenib) and, to a lesser extent, with monoclonal antibodies. In addition, the oncogenic or tumor suppressor potential of this signaling pathway has aroused the interest of the scientific community in evaluating p38MAPK as a novel target for cancer therapy. In this review, we will summarize the role of p38MAPK in chemotherapy as well as the potential that p38MAPK inhibition can bring to cancer therapy. All the evidences suggest that p38MAPK could be a double-edged sword and that the search for the most appropriate candidate patients, depending on their pathology and treatment, will lead to a more rational use of this new therapeutic tool.

## Introduction

The p38MAPK pathway belongs to the group of stress-activated kinases, composed by p38MAPK (with four isoforms) and JNK (with, at least, 10 isoforms grouped into JNK1, 2, and 3) (reviewed in Kyriakis and Avruch, 2012). This MAPK signaling pathway, initially discovered as a stress response mechanism and as an osmolality sensor (Han et al., 1994), resulted to be implicated in different human pathologies such as rheumatoid arthritis (Clark and Dean, 2012), or neurodegenerative diseases (Corrêa and Eales, 2012) among others. The involvement of p38MAPK in cancer has been widely described (for review see Wagner and Nebreda, 2009). However, its role as an oncogene or tumor suppressor is unclear. In some experimental models, it has been shown to have tumor suppressor properties, for example through the control of oxidative stress (Dolado et al., 2007) while in others, it is clearly associated to survival and oncogenesis (Alvarado-Kristensson et al., 2004; Comes et al., 2007). Indeed, this dual role has been recently demonstrated in colorectal cancer, where it is described as a tumor suppressor in early stages, but in later stages it is required for cell survival with oncogenic traits (Gupta et al., 2014). In any case, it is clear that, regardless of p38MAPK dual characteristic as a possible oncogene or tumor suppressor, this signaling pathway is implicated in different types of tumors (for review see Koul et al., 2013).

Moreover, p38MAPK has been connected with apoptotic cell death. In fact, key molecules in the apoptotic onset—such as Bcl2 superfamily members or p53—have been shown to be substrate of this MAPK (Sanchez-Prieto et al., 2000; Cai et al., 2006). This observation prompted to consider p38MAPK as a key player in the response to chemotherapy, considering that apoptosis is the main mechanism of cell death associated to it. Therefore, the study of p38MAPK in the last decades was focused in its implication in the response to chemotherapy. In the following pages, we will show some examples of how p38MAPK can modulate the response to several chemotherapeutic agents that are currently used in the clinical practicum. This includes conventional chemotherapeutic agents and novel therapeutic approaches like tyrosine kinase inhibitors or monoclonal antibodies. Although all the members of the p38MAPK family have been related to cancer and its therapy in different experimental models (Pillaire et al., 2000; Cerezo-Guisado et al., 2011; Hou et al., 2012; Del Reino et al., 2014; O'Callaghan et al., 2015; Zur et al., 2015), in this paper we will focus onto p38MAPKα as the most studied and representative member of the family.

## p38MAPK and cisplatin

Cisplatin (CDDP) is one of the most widely used drugs in cancer therapy and probably the best example of the connection between p38MAPK and conventional chemotherapy (Brozovic and Osmak, 2007). p38MAPK was related to the cellular response to CDDP through the c-Abl signaling pathway (Pandey et al., 1996). Moreover, p38MAPK mediates activation of p53 in response to CDDP suggesting a role in resistance (Sanchez-Prieto et al., 2000). In fact, this molecule can be activated by other platinum-based compounds even without toxicity as in the case of Trans-platin (Hernández Losa et al., 2003). However, the relationship between CDDP and p38MAPK has two sides: one is related to resistance and the other, to sensitivity. Initially, the role as a determinant of resistance was proposed mainly based on the inhibition of p38MAPK in different experimental models (Mansouri et al., 2003; Brozovic et al., 2004; Baldwin et al., 2006). Indeed, several substrates of p38MAPK seem to be implicated in resistance to CDDP such as p18^Hamlet^ (Cuadrado et al., 2007) or ATF3 (St Germain et al., 2010). Furthermore, hyperactivation of p38MAPK through MKK3, that renders a non-functional pathway, has been correlated with resistance (Galan-Moya et al., 2011). However, recent evidences demonstrated a sensitizing role for the inhibition of p38MAPK both *in vivo* and *in vitro* through the production of Reactive Oxygen Species, which promotes the activation of the JNK pathway and thus sensitizing human tumor cells to CDDP-associated apoptosis (Pereira et al., 2013). In this regard, it has been proposed that certain p38MAPK downstream molecules (Hsp27, ERCC1, or Fox3a) can mediate sensitivity associated to p38MAPK inhibition (Planchard et al., 2012; Germani et al., 2014; Liu et al., 2016). In addition, inhibition of p38MAPK could also facilitate sensitivity in specific contexts as in the case of the presence of the adenoviral protein E1A (Cimas et al., 2015). Nonetheless, new platinum-based compounds have been developed and, some of them, for instance, Satraplatin or Picoplatin are in clinical use, (Doshi et al., 2012; Hamilton and Olszewski, 2013), but there is no clue about the role of p38MAPK. Only in the case of Monoplatin, a non-DNA binding platinum-based compound only used in cell culture so far, cell-type specific activation of p38MAPK has been demonstrated, but with no effect in terms of resistance/sensitivity (García-Cano et al., 2015). In conclusion, the dual role of p38MAPK as a mechanism of resistance/sensitivity to CDDP could be related to specific features such as cell type, downstream molecules or other signaling pathways.

## p38MAPK and cytarabine

Cytarabine -also known as ara-C-, a deoxycytidine analog, is an antileukemic agent that incorporates into DNA promoting strand breaks (Fram and Kufe, 1982; Major et al., 1982). Cytarabine promotes both cell death and differentiation in leukemia cells (Grant et al., 1996). It has been demonstrated that Cytarabine induces apoptosis through p38MAPK and JNK in a c-Abl dependent fashion (Saleem et al., 1995; Pandey et al., 1996). In this sense, it has been suggested that Cytarabine-induced apoptosis can be blocked by the specific inhibition of p38MAPK in HL-60 cells, (Stadheim et al., 2000). Moreover, in chronic myeloid leukemia cells, the constitutive activation of p38MAPK by BCR/Abl renders a Cytarabine-insensitive phenotype (Sánchez-Arévalo Lobo et al., 2005), suggesting a role for p38MAPK in the resistance to Cytarabine. Interestingly, a study in acute myeloid patients treated with Cytarabine and Daunorubicin showed that active p38MAPK and JNK correlate with cell death in chemosensitive patients (Maha et al., 2009). Therefore, most of the evidences support that the lack of functionality in p38MAPK could mediate a resistant phenotype to Cytarabine.

## p38MAPK and gemcitabine

Gemcitabine is a deoxycytidine analog, widely used for treating different carcinomas such as pancreatic, bladder, breast and non-small cell lung cancer (Gesto et al., 2012). Cell death associated to Gemcitabine has been related to the p38MAPK pathway (Nakashima et al., 2011; Liu et al., 2014). Indeed, a study performed in human urothelial carcinoma sub-lines with acquired Gemcitabine resistance showed a marked repression in p38MAPK activity and an increase in gemcitabine sensitivity when expression of p38MAPK was forced (Kao et al., 2014). It has also been described that Gemcitabine induces phosphorylation of p38MAPK substrates like Hsp27 that could be mediating acquired resistance in pancreatic cancer cell lines (Kang et al., 2015). In addition, there are evidences showing how the p38MAPK/MK2 stress response pathway is required for the cytotoxic effect of Gemcitabine in osteosarcoma and pancreatic cancer cells (Köpper et al., 2013, 2014). However, the use of p38MAPK as a putative biomarker for the response to Gemcitabine is still unexplored and, in the few studies performed so far, results are disappointing as in the case of platinum resistant recurrent ovarian cancer (Klotz et al., 2008). Therefore, all the evidences support a definitive role for p38MAPK and different p38MAPK substrates as key players in Gemcitabine response, in which blockage of p38MAPK seems to be a key mechanism of resistance that still needs to be more investigated for future clinical use.

## p38MAPK and 5-FLUOROURACIL

5-Fluorouracil (5FU) irreversibly inhibits thymidylate synthase. It is widely used in the treatment of solid tumors such as breast, colorectal, stomach, pancreatic, oesophageal and skin cancers (Longley et al., 2003). The implication of the p38MAPK signaling pathway in the response to 5FU has been studied since late 90's (Wu et al., 1998). 5FU-associated cell death works through the induction of p53-dependent apoptosis (Mariadason et al., 2003). The role of p38MAPK in terms of resistance or sensitivity is still not clear. On the one hand, a report demonstrated that p38MAPK inhibition renders a blockage of p53-dependent apoptosis allowing an autophagic response that mediates resistance (de la Cruz-Morcillo et al., 2012). On the other hand, other reports support that the inhibition of p38MAPK and the subsequent effect onto Hsp27 can promote sensitivity to this drug (Yang et al., 2011; Matsunaga et al., 2014). In this regard, the high dose of the inhibitor used, i.e., 50 μM SB203580, and the lack of a genetic approach suggest that the inhibition of other molecules, in addition to p38MAPK, could be implicated. Finally, the antiangiogenic properties through the induction of Thrombospondin-1, the mucositis and the release of pro-inflammatory cytokines associated to 5FU are also mediated by p38MAPK (Elsea et al., 2008; Zhao et al., 2008; Gao et al., 2014). In summary, most of the evidences support a role for p38MAPK in 5FU-based therapy in terms of therapeutic response, as well as in other aspects, that need to be fully elucidated for its clinical applications.

## p38MAPK and targeted therapy

The idea of a targeted therapy has become a gold standard in cancer therapy, being Imatinib (Gleevec, STI571), a specific inhibitor of BCR/Abl (Druker et al., 1996), its first example. Later, it was shown how the activation of p38MAPK was directly implicated in the survival of KT-1 cells in response to Imatinib (Parmar et al., 2004). Almost at the same time, it was also demonstrated how p38MAPK activation was a key event in the differentiation effect of Imatinib in K562 cells, but with no effect onto cell viability (Kohmura et al., 2004) probably due to the lack of effect of p38MAPK onto caspase activation (Jacquel et al., 2007). Indeed, in a resistant model by continuous co-culturing with this drug, p38MAPK showed a lack of implication in the acquired resistance phenotype (Aceves-Luquero et al., 2009). In fact, it has been proposed that the connection between Abl and p38MAPK is not related to the tyrosine kinase activity of Abl (Galan-Moya et al., 2008), indicating that the activation of p38MAPK in the presence of Imatinib could be a secondary event, rather than a direct activation by this compound. Nonetheless, it has been shown how p38MAPK could be implicated in the response to second generation of BCR/Abl inhibitors as in the case of Dasatinib (Dumka et al., 2009) with a direct implication in some of the side effects like hepatotoxicity (Yang et al., 2015). However, the role of p38MAPK in the response to other BCR/Abl inhibitor like Nilotinib remains unclear and has merely been investigated as side studies in other experimental models as sarcoma or colorectal cancer derived cell lines (Villar et al., 2012; Rey et al., 2015) or in combination with other drugs (Bucur et al., 2013).

But the role of p38MAPK is not restricted to the response to BCR/Abl inhibitors. For example, the multikinase inhibitor Sorafenib (BAY 43-9006), originally described as a Raf inhibitor, used in the treatment of several pathologies such as hepatocarcinoma or renal cell carcinoma among others (Wilhelm et al., 2008), has been also related to p38MAPK. In this sense, it has been shown how the activation of p38MAPK can be a mechanism of resistance and a novel biomarker for Sorafenib-based therapy in hepatocellular carcinoma (Rudalska et al., 2014). Indeed, it has been recently proposed how the combination with mTOR inhibitors can potentiate the effect of Sorafenib in malignant pleural mesothelioma through p38MAPK-dependent apoptosis (Pignochino et al., 2015). Finally, in the case of an EGFR inhibitor, known as Iressa (Genfitinib), early evidences demonstrate a lack of effect on the p38MAPK signaling pathway in different experimental models (Höpfner et al., 2003; Kokubo et al., 2005). However, in certain leukemic and intestinal epithelial cells, p38MAPK activation was observed (Moon et al., 2007; Sheng et al., 2007), but no definitive effect in terms of resistance/sensitivity has been reported. Nonetheless, the role of p38MAPK in Iressa-based therapy could be related to the combination with other compounds. For example, in the case of curcumin, p38MAPK attenuates the adverse gastrointestinal effects (Lee et al., 2011), or, in combination with metformin, p38MAPK inhibition can potentiate the effect of Iressa (Ko et al., 2013). Therefore, the full role of p38MAPK in Iressa-based therapy still needs to be elucidated.

Finally, another targeted therapy modality is the use of monoclonal antibodies (Dienstmann et al., 2012; Henricks et al., 2015). In this sense, the literature regarding p38MAPK is much less abundant. For example, for Trastuzumab, an antibody against Her2/neu, the few studies where p38MAPK is considered support a direct role for this MAPK in resistance to this antibody (Yong et al., 2013; Donnelly et al., 2014). In the case of EGFR-directed antibody Cetuximab, the main connection with p38MAPK has been established in combination with Oxaliplatin (Santoro et al., 2015), in which Cetuximab blocks Oxaliplatin-triggered p38-dependent apoptosis, explaining the lack of success for this combination in some patients of colorectal cancer. Finally, in the case of Bevacizumab, a monoclonal antibody against VEGF, there is no evidence linking directly MAPK to this antibody and only as a side study in mesenchymal stem cells, melanoma and pancreatic carcinoma cells s (De Luca et al., 2012; Jiang et al., 2012).

## Future directions: p38MAPK inhibitors

The development of new and more specific inhibitors is a critical step in a future therapy based on p38MAPK inhibition. Several pathologies are considered susceptible for this strategy. In the case of chronic obstructive pulmonary disease (COPD), the use of p38MAPK inhibitor PH-797804 (Selness et al., 2011) turned out to have preliminary positive results in healthy volunteers and in patients (MacNee et al., 2013; Singh et al., 2015). In addition, the p38MAPK inhibitor GW856553 (Losmapimod) succeeded to ameliorate exacerbations in patients suffering COPD with low eosinophil levels (Marks-Konczalik et al., 2015). Not only does Losmapimod bear a therapeutic potential for COPD, but also it has been tested against coronary artery diseases such as acute myocardial infarction through its anti-inflammatory activity (O'Donoghue et al., 2015). This specific p38MAPK inhibitor has become an alternative treatment to several disorders derived from acute coronary syndrome (thoroughly reviewed in Kragholm et al., 2015). Many other diseases—atherosclerosis, Alzheimer's disease, depression, immunological diseases and so on—are sought to be treated by the use of p38MAPK inhibitors (Supplementary Material 1). However, although in some pathologies, namely rheumatoid arthritis, the inhibition was considered as a promising therapeutic approach (McLay et al., 2001) clinical results are disappointing (Genovese et al., 2011), thus suggesting the complexity of the biological function for p38MAPK.

Regarding cancer therapy, p38MAPK inhibition by itself is also considered a promising target (Igea and Nebreda, 2015). In fact, in some cases like in colorectal cancer, experimental evidences support this novel therapeutic approach (Gupta et al., 2015), and it has also been reported recently that p38MAPK inhibition overcomes the resistance to compounds like Birinapant in primary acute myeloid leukemia (Lalaoui et al., 2016). Ongoing clinical trials are showing the safety of the p38MAPK inhibitors as in the case of Ralimetinib (Patnaik et al., 2016) or ARRY-614 (Garcia-Manero et al., 2015). Currently, there are 59 clinical trials in different stages, including healthy volunteers in phase I studies, in which p38MAPK is evaluated as a potential biomarker or target for different diseases such as arthritis or heart and pulmonary disease among others^1^. From this wide range, 14 of them are related to cancer and only in 7 of these ongoing clinical trials, p38MAPK is regarded as a target in cancer treatment, by using specific inhibitors alone or in combination with other therapeutic agents (Table 1). Therefore, it seems logical to look for adequate candidates that might be benefited with the use of p38MAPK inhibitors. The mutation ratio in p38MAPK or MAP2K3/6 in human cancer is extremely low^2^ (i.e., Pritchard and Hayward, 2013), suggesting that the implication of an active p38MAPK in cancer is due to the pathologic context of the tumor rather than a genetic alteration in the MAPK or MAP2K able to render a constitutive pathway. One possibility could be the evaluation of the expression / activity levels of the different components of this signaling pathway by immunohistochemistry, as it has been shown for different pathologies, allowing us to choose the best candidate patient for a p38MAPK inhibition-based therapy. It is important to be as careful as possible due to the dual role of this signaling pathway in cancer. For example, MAP2K3 (MKK3) has been proposed as a tumor suppressor in breast cancer (MacNeil et al., 2014) thus suggesting that the inhibition of p38MAPK signaling pathway could be counterproductive in this type of tumors. However, recent evidences show how inhibition of this same molecule is a novel therapeutic approach in different experimental models (Baldari et al., 2015). Therefore, the antitumoural activity associated to p38MAPK could also be used in combination with current therapies in order to potentiate its effects, increasing the number of putative patients that can be benefited from the use of p38MAPK inhibitors. Nonetheless, we should not discard the potential side effects of p38MAPK inhibition as a mechanism of drug resistance as well as the loss of its tumor suppressor potential.

**Table 1:** **p38MAPK inhibitors used in ongoing clinical trials in which p38MAPK is used as a target in cancer therapy**.

**Inhibitor**	**Pathology**	**Combination (if Available)**	**Trial ID**	**Date**	**Phase**
Ralimetinib (LY2228820 dimesylate)	Metastatic breast cancer (MBC)	Tamoxifen	NCT02322853	2014	II
LY2228820	Recurrent Ovarian Cancer	–	NCT01663857	2012	I/II
LY3007113	Advanced Cancer (Either Solid Tumors or Lymphomas)	–	NCT01463631	2011	I
LY2228820	Advanced Cancer (Either Solid Tumors or Lymphomas)	–	NCT01393990	2011	I
LY2228820	Glioblastoma	TMZ and Radiotherapy	NCT02364206	2015	I/II
ARRY-614	Myelodysplastic Syndrome	–	NCT00916227	2009	I
ARRY-614	Myelodysplastic Syndrome	–	NCT01496495	2011	I

*Source: https://clinicaltrials.gov/ct2/results?term=p38+inhibitor+cancer&Search=Search (as accessed in April 2016)*.

To sum up, the use of p38MAPK as a potential target for cancer therapy should be carefully considered based on the pathology and the therapy used in order to avoid adverse effects, as could be the generation of resistances or more aggressive phenotypes. The deep understanding of the role of p38MAPK in cancer therapy can lead to a new era of better prognosis and personalized therapy in which p38MAPK inhibitors could be a cornerstone.

## Author contributions

JG contributed in the Introduction and in the “Future Directions” section. He intervened in editing tasks and in gathering information for Supplementary Material 1 and Table 1 and thoroughly proofread the manuscript for its submission. OR collaborated in the composition of “p38MAPK and Cytarabine” section, provided proofreading comments and helped in the edition. FC collaborated in the composition of “p38MAPK and Cisplatin” section, provided proofreading comments and helped in the edition. RP collaborated in the composition of “p38MAPK and Gemcitabine and 5-Fluorouracil” sections. MO collaborated in the composition of “Targeted Therapies” section. DF collaborated in the composition of Supplementary Material 1. RS designed and coordinated the whole review, collaborated composing all the sections, and thoroughly proofread the manuscript for its submission.

## Funding

This work was supported by grant from Fundación Leticia Castillejo Castillo, and MINECO (SAF2015-64215-R).

### Conflict of interest statement

The authors declare that the research was conducted in the absence of any commercial or financial relationships that could be construed as a potential conflict of interest.

## Footnotes

^1^https://clinicaltrials.gov/

^2^http://cbioportal.com
