# Supplementary material for: p38MAPK and Chemotherapy: We Always Need to Hear Both Sides of the Story
Source: Front Cell Dev Biol. 2016 Jun 30;4:69. doi: 10.3389/fcell.2016.00069 (PMC4928511; doi:10.3389/fcell.2016.00069)
Supplement: Supplementary file 1 [file Table1.DOCX]

| Inhibitor | Pathology | Combination (if Available) | Trial ID | Date | Phase |
| --- | --- | --- | --- | --- | --- |
| p38 inhibitor (4) | Rheumatoid arthritis | Methothrexate | NCT00316771 | 2006 | II |
| p38 inhibitor (4) | Rheumatoid arthritis | - | NCT00303563 | 2006 | II |
| PH-797804 | Rheumatoid arthritis | - | NCT00383188 | 2006 | II |
| PH-797804 | Rheumatoid arthritis | Methothrexate | NCT00620685 | 2008 | II |
| ARRY-371797 | Postoperative pain (third molar extraction) | Celecoxib | NCT00663767 | 2008 | II |
| ARRY-371797 | Postoperative pain (third molar extraction) | - | NCT00542035 | 2007 | II |
| ARRY-371797 | LMNA-Related Dilated Cardiomyopathy | - | NCT02351856 | 2015 | II |
| ARRY-371797 | LMNA-Related Dilated Cardiomyopathy | - | NCT02057341 | 2014 | II |
| ARRY-371797 | Osteoarthritis of the Knee | Oxycodone hydrochloride (HCl) | NCT01366014 | 2011 | II |
| ARRY-371797 | Ankylosing Spondylitis | - | NCT00811499 | 2008 | II |
| ARRY-371797 | Rheumatoid Arthritis | - | NCT00729209 | 2008 | I |
| VX-745 | Alzheimer’s Disease | - | NCT02423200 | 2015 | II |
| SB681323 | Neuropathic Pain | - | NCT00390845 | 2006 | II |
| VX-745 | Alzheimer’s Disease | - | NCT02423122 | 2015 | II |
| BMS-582949 | Atherosclerosis | Atorvastatin, Statin | NCT00570752 | 2007 | II |
| GW856553 (Losmapimod) | Acute Coronary Syndrome | - | NCT02145468 | 2014 | III |
| GW856553 (Losmapimod) | Neuropathic Pain | - | NCT00969059 | 2009 | II |
| GS856553 | Neuropathic Pain (Lumbosacral Radiculopathy) | - | NCT01110057 | 2010 | II |
| SB-681323 | Rheumatoid Arthritis | Methothrexate | NCT00419809 | 2005 | I |
| VX-702 | Rheumatoid Arthritis | - | NCT00395577 | 2006 | II |
| VX-702 | Rheumatoid Arthritis | - | NCT00205478 | 2005 | II |
| GW856553 (Losmapimod) | Major Depressive Disorder | - | NCT00976560 | 2009 | II |
| SB-681323 | Acute Lung Injury | - | NCT00996840 | 2009 | II |
| GW856553 (Losmapimod) | Chronic Obstructive Pulmonary Disease | - | NCT01541852 | 2012 | II |
| GW856553 (Losmapimod) | Acute Coronary Syndrome | - | NCT00910962 | 2009 | II |
| SB-681323 | Coronary Heart Disease | - | NCT00291902 | 2006 | II |
| SB-681323 | Chronic Obstructive Pulmonary Disease | - | NCT00564746 | 2007 | I |
| GW856553 (Losmapimod) | Acute Coronary Syndrome | Moxifloxacin | NCT01756495 | 2013 | I |

Supplementary Material 1. p38MAPK inhibitors used in the ongoing clinical trials in which p38 is used as a target in pathologies other than cancer.

Source: <https://clinicaltrials.gov/ct2/results?term=p38+inhibitor&Search=Search> (accessed in april 2016)
